# Navigating Anesthesia in Fragile Patients: A Case Report on Esophageal Dilation in a 9‐Year‐Old Patient With Epidermolysis Bullosa

**DOI:** 10.1155/cria/5513410

**Published:** 2026-05-07

**Authors:** Yumna Abdulmalek Bokhari, Rayan Mohammed Bakhreba, Mohammed Abdalmajid Nyaz

**Affiliations:** ^1^ Department of Anesthesia, King Fahad Armed Forces Hospital, Jeddah, Saudi Arabia, kfafh.org

**Keywords:** airway management, difficult airway, epidermolysis bullosa, esophageal dilatation, pediatric anesthesia

## Abstract

Epidermolysis bullosa (EB) is a rare genetic disorder causing fragile skin and mucous membranes, leading to blistering from minor trauma. Managing anesthesia in EB patients is challenging due to their delicate airways, limited mobility, and need for repeated surgeries. We describe the perioperative management of a 9‐year‐old girl with dystrophic EB undergoing esophageal dilation. Preoperative assessment showed limited mouth opening, poor dentition, restricted neck movement, and extensive scarring, requiring a difficult airway approach. Anesthesia was induced intravenously, and intubation was successful on the first attempt using a lubricated video laryngoscope and cuffed tube, avoiding trauma. Monitoring was adapted to eliminate adhesives, and padding was used to prevent new blisters. The procedure was completed without complications, and extubation was performed while the patient was awake. This highlights the importance of preparation, teamwork, and tailored anesthetic techniques to ensure safety in EB patients, helping reduce risks and guiding management.

## 1. Introduction

Epidermolysis bullosa (EB) is a rare, inherited group of disorders characterized by mechanical fragility of the skin and mucous membranes, resulting in blistering and erosion after minimal trauma. Anesthetic management is complex due to fragile airway structures, limited mouth opening, and the risk of new skin or mucosal injury from monitoring and airway manipulation. Several case reports and series have described various anesthetic strategies in patients with EB, including careful airway planning using videolaryngoscopy or fiberoptic techniques, modified nonadhesive monitoring, and gentle handling to avoid trauma during both pediatric and adult procedures such as esophageal dilatation and dental surgery, emphasizing multidisciplinary care to reduce perioperative complications [1–4].

The main challenge is ensuring safe airway management, as patients with EB have fragile airway structures that make intubation and ventilation difficult. Their delicate skin also requires careful handling to prevent blistering and injury during procedures and postoperative care.

We report a case of anesthetic management in a pediatric patient, with epidermolysis bullosa dystrophica (DEB), undergoing esophageal dilatation, highlighting airway management and skin‐protective strategies.

## 2. Case Presentation

This case involves a 9‐year‐old female, weighing 16 kg, with dystrophic EB, associated with dysphagia, failure to thrive, and chronic constipation, scheduled for esophageal dilation under general anesthesia. Diagnosed at birth, her condition worsened 3 years ago, requiring esophageal dilations approximately every 3 months. She underwent the same procedure 3½ months ago at our hospital without any anesthetic complications. At that time, it was her first procedure at our facility and the first DEB case we managed; prior to this, she had been followed in Riyadh. She was born to nonconsanguineous parents with no family history of the condition.

**FIGURE 1:**
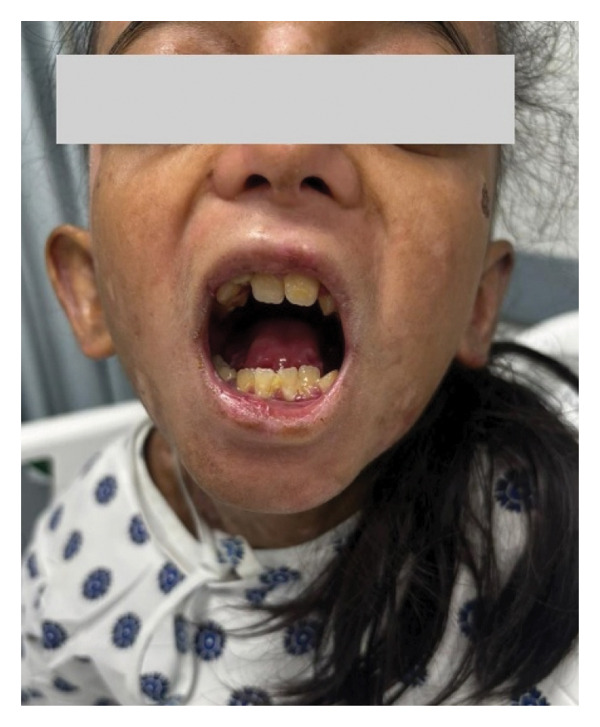
Intraoral view of the patient showing dental abnormalities and limited mouth opening.

She presented with solid dysphagia and distal esophageal stenosis. Airway assessment revealed limited mouth opening (< 6 cm), restricted neck mobility, and oral changes, including poor dentition; Mallampati classification could not be assessed (Figure 1). Examination showed generalized scars, extensive blisters and erosions, healed wounds, and syndactyly of fingers and toes. Preoperative evaluation was performed, risks explained, and parental consent obtained.

The operating room was prepared for a difficult airway with a trolley containing various airway devices, endotracheal tubes, LMAs, bougie, and emergency medications. All pressure areas with blisters were protected, and a 22G intravenous cannula, previously placed by the primary nurse, was inspected, flushed, and confirmed to be secure and patent prior to induction. Preoperative sedation included 1 mg midazolam.

**FIGURE 2:**
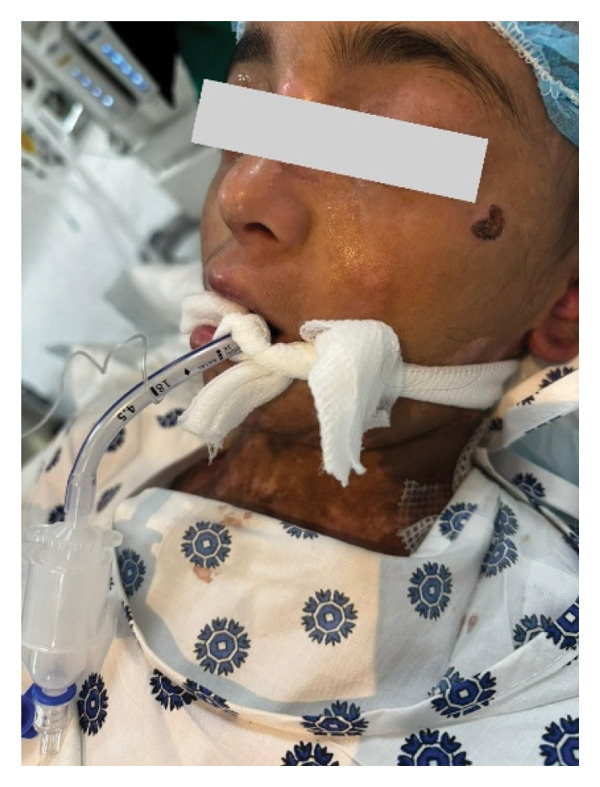
Patient is intubated with a lubricated tie secured around the neck.

Intraoperatively, the patient was carefully positioned on soft padding. Monitoring included pulse oximetry, which was already present on the patient’s finger before receiving, and a padded noninvasive blood pressure cuff, which was utilized solely to obtain a baseline blood pressure reading and was not used for repetitive monitoring to reduce the risk of skin trauma. The temperature probe was not applied intraoperatively as it was a short procedure; only baseline temperature was recorded in the receiving area, but a warmer device was applied onto the patient body before induction to prevent heat loss. Application of ECG electrodes was avoided due to the presence of multiple blisters and open wounds on the patient’s chest, which posed a risk of further skin injury. The patient was also cautious about exposing the chest area. Alternative approaches were considered, including trimming the adhesive portion of the electrodes to allow contact only with the hydrogel center or placing gauze around the adhesive edges so that only the central gel contacted the skin. However, ECG monitoring was ultimately omitted as the patient had no known cardiac involvement, the procedure was expected to be short, and this decision was made in accordance with the specific anesthetic protocol. Preoxygenation was performed with a lubricated face mask. Anesthesia was induced with 2% lidocaine total 20 mg, fentanyl 30 mcg (10 mcg/mL dilution), 1% propofol 70 mg, and rocuronium 10 mg (10 mg/mL). Atraumatic single‐attempt intubation was performed using a lubricated McGrath laryngoscope and 4.5 cuffed ETT. The tube was secured with a lubricated tie, eyes were protected, and since it is a shared airway, glycopyrrolate 80 mcg was administered prophylactically to reduce airway secretions, with the aim of avoiding suctioning prior to extubation and thereby minimizing the risk of airway trauma. Hydrocortisone 40 mg and granisetron 0.2 mg were administered (Figure 2). Endotracheal tube cuff pressure was not monitored; however, we applied titrated volume and pressure to the cuff until appropriate tidal volume was achieved. Maintenance was with 2% sevoflurane in 50% air/O_2_, along with 150 mL normal saline and 250 mg paracetamol.

The 40‐minute procedure concluded with smooth extubation after 60 mg IV sugammadex. To avoid agitation during emergence, we administered paracetamol intraoperatively for proper analgesia and glycopyrrolate to avoid excessive suctioning. The patient was monitored for 1 h in the post‐anesthesia care unit before transferring to the pediatric ward (Figures​ 3, 4, and 5).

**FIGURE 3:**
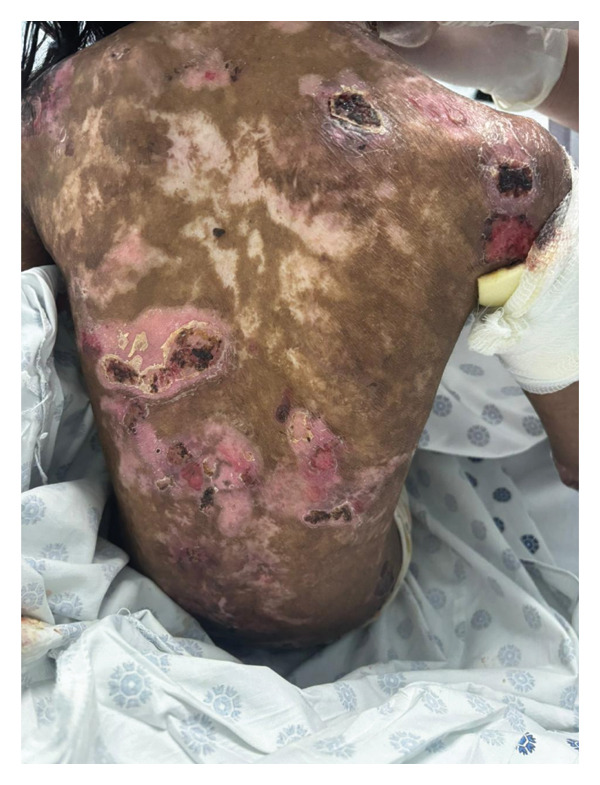
Extensive blistering and eroded skin lesions prior to antiseptic dressing.

**FIGURE 4:**
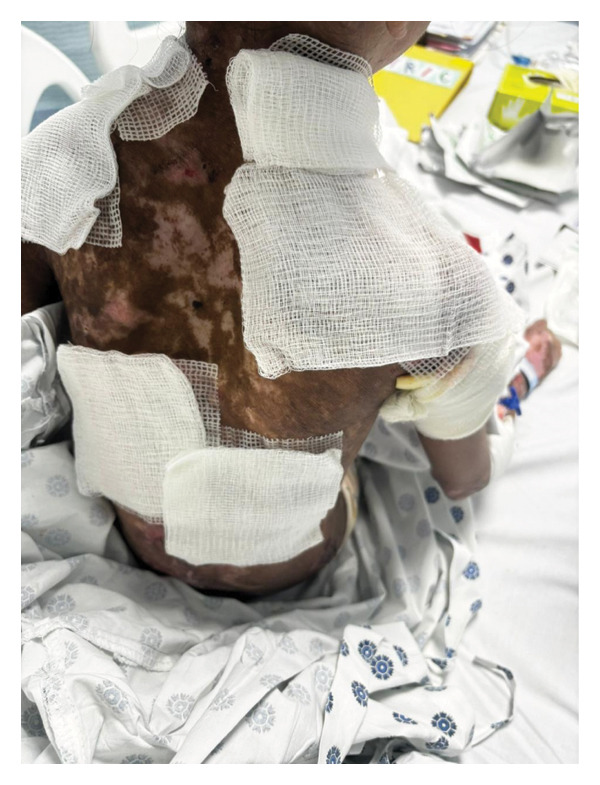
Open blistered areas padded with chlorhexidine antiseptic dressing coated with ointment and covered with gauze to prevent skin trauma.

**FIGURE 5:**
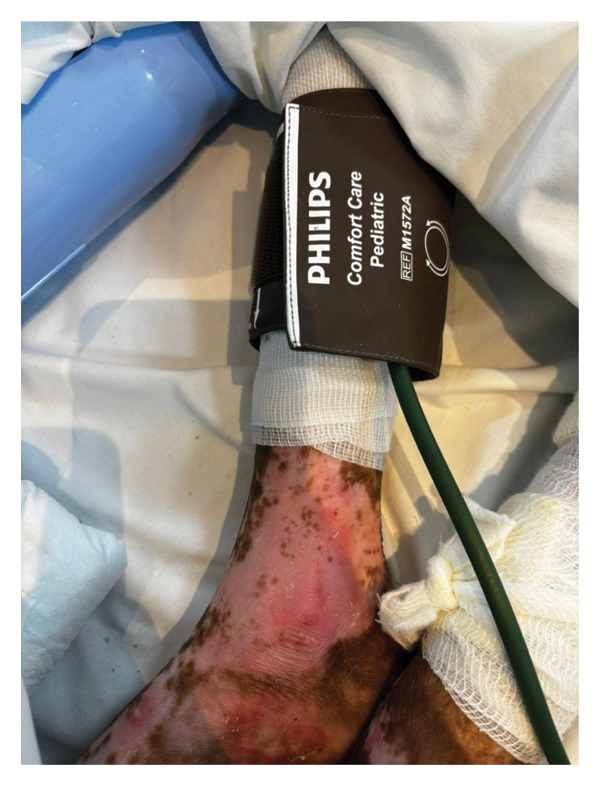
Blood pressure cuff placed on top of soft padding.

Table 1 summarizes the perioperative recommendations implemented for this patient, which may guide anesthetic management in similar cases of DEB.

**TABLE 1:** Perioperative recommendations for anesthetic management in pediatric dystrophic epidermolysis bullosa (DEB).

Clinical aspect	Recommendation	Rationale
Airway assessment and management	Perform thorough preoperative airway evaluation; prepare videolaryngoscope, fiberoptic scope, stylets, and smaller lubricated cuffed ETT	Anticipates difficult airway due to microstomia, poor dentition, and limited neck mobility; minimizes airway trauma
Endotracheal tube fixation	Secure tube with lubricated ties instead of adhesive tape	Prevents skin and mucosal injury
Airway secretions	Administer prophylactic glycopyrrolate	Reduces need for suctioning, minimizing airway trauma
Monitoring: blood pressure	Use padded noninvasive cuff for baseline reading only; avoid repetitive cycling	Reduces risk of skin trauma in fragile areas
Monitoring: ECG	Avoid adhesive electrodes; consider hydrogel‐only contact or gauze padding if needed	Prevents blistering and skin injury; may omit if no cardiac issues and procedure is short
Positioning and pressure points	Pad all open blisters, scars, and pressure points with nonadherent antiseptic dressings and gauze	Prevents shear injury, skin tears, and pressure ulcers
Skin protection	Avoid adhesive devices; lubricate any necessary contact surfaces	Minimizes friction and trauma to fragile skin
Induction and maintenance	Use IV induction agents, perform gentle handling, and avoid repeated airway manipulation; maintain with standard anesthetics as tolerated	Reduces risk of new blisters or mucosal injury
Extubation	Extubate awake when possible	Minimizes airway trauma and aspiration risk
Analgesia and antiemetic	Administer perioperative analgesics, steroids, and antiemetics as indicated	Reduces postoperative pain, edema, and nausea while minimizing interventions that could traumatize skin or mucosa

Written informed consent for conducting and publication of the study was obtained from the patient’s mother. Ethical approval was obtained from the King Fahad Armed Forces Hospital research center.

## 3. Discussion

DEB presents notable anesthetic challenges due to the inherent skin and mucosal fragility, airway involvement, and the frequent need for repeated procedures such as esophageal dilatation. Careful perioperative planning is crucial to minimize trauma and improving outcomes. Our case shows that with careful preparation, anesthesia can be safely administered even with extensive skin lesions, airway restrictions, and systemic involvement.

Preoperative assessment should account for the potential of a difficult airway, as patients often present with microstomia, dental problems, contractures, and mucosal scarring. In our patient, limited mouth opening, poor dentition, and restricted neck movement necessitated readiness with a videolaryngoscope, stylet, and a smaller, lubricated, cuffed tube. This approach aligns with multiple reports emphasizing video‐assisted intubation or fiberoptic techniques as safer options compared to traditional laryngoscopy in DEB patients [5, 6].

General anesthesia is often used for esophageal dilatation and, when properly managed, is regarded as safe. A retrospective review of 129 anesthetic procedures in 32 patients with EB found that, with proper precautions, standard anesthetic techniques can be used with only minor complications [7].

Recently, a large case series from a tertiary referral center analyzed 202 anesthetics in 37 EB patients and found low adverse outcome rates when a structured care pathway was used, with only 4% experiencing new skin or mucosal injuries and no major complications [8]. Skin protection during anesthesia is equally important. Adhesives should be avoided, and monitoring devices need adjustments to prevent shear injuries. In our case, blood pressure cuffs were padded with gauze, pulse oximetry was directly connected to an existing probe, and ECG electrodes were omitted to prevent blistering. These measures reflect published recommendations and institutional protocols that recommend nonadhesive monitoring, thorough padding of pressure points, and lubrication of airway and monitoring devices [5, 8, 9].

Securing the endotracheal tube with lubricated ties instead of adhesive tape has been shown to decrease mucosal trauma and was used in this case [6, 10]. Esophageal dilatation is one of the most common procedures in children with DEB, and case reports indicate that it can be safely performed under general anesthesia with protective modifications. Kandemir et al. reported safe anesthetic management for esophageal dilatation in an 18‐year‐old with DEB, using strategies such as lubrication, careful intubation, and protective padding [11]. In our case, the patient was induced intravenously, intubated with rocuronium, and maintained on sevoflurane. Although anesthetic management of EB has been previously reported, this case uniquely integrates airway strategy, procedure‐specific risk mitigation for esophageal dilatation, and comprehensive perioperative skin protection in a pediatric patient with DEB and predictors of a difficult airway, demonstrating safe first‐attempt videolaryngoscopic intubation. Analgesics and antiemetics were administered, along with perioperative steroids to reduce airway edema, all consistent with guidelines for safe anesthesia in EB [8, 9]. Extubation was performed when the patient was fully awake, as awake extubation minimizes mucosal trauma and aspiration risk and is generally recommended for this population [8].

This case demonstrates that safe anesthetic management in pediatric patients with dystrophic EB undergoing esophageal dilatation is achievable with meticulous planning and a multidisciplinary approach. Careful airway assessment, gentle handling, avoidance of adhesives, modified monitoring, and protective positioning were key to minimizing perioperative complications.

Awareness of how to care for fragile patients is essential for providing optimal management, with an emphasis on multidisciplinary care with anesthesiologists planning and executing atraumatic airway management, surgeons performing procedure‐specific interventions, and nursing staff ensuring gentle handling and pressure‐point padding, all coordinated to minimize trauma and complications.

Based on this case, several aspects of general anesthesia management in patients with EB require special consideration. Careful preoperative airway planning and early use of videolaryngoscopy are essential due to anticipated difficulty. Atraumatic techniques, including adequate lubrication and gentle handling, should be consistently applied to minimize mucosal and skin injury.

Additionally, modified monitoring strategies should be optimized to balance patient safety with the need to avoid adhesives. Finally, given the need for repeated procedures, establishing standardized protocols and a multidisciplinary approach is crucial to improve safety and consistency in future anesthetic management.

## Funding

No funding was received for this manuscript.

## Ethics Statement

This study was approved by the Research Ethics Committee of Armed Forces Hospital–Jeddah (Application No. 2025‐65).

## Consent

Written informed consent for participation and photography was obtained from the patient’s mother on the day of the procedure, after a thorough explanation of all steps of the procedure and the study.

## Conflicts of Interest

The authors declare no conflicts of interest.

## Data Availability

The data that support the findings of this study are available from the corresponding author upon reasonable request.
